# Macrolides: From Toxins to Therapeutics

**DOI:** 10.3390/toxins13050347

**Published:** 2021-05-12

**Authors:** Kiersten D. Lenz, Katja E. Klosterman, Harshini Mukundan, Jessica Z. Kubicek-Sutherland

**Affiliations:** Physical Chemistry and Applied Spectroscopy, Chemistry Division, Los Alamos National Laboratory, Los Alamos, NM 87545, USA; kiersten@lanl.gov (K.D.L.); katja@lanl.gov (K.E.K.); harshini@lanl.gov (H.M.)

**Keywords:** macrolide, toxin, antibiotic, antifungal, antiviral, antiparasitic, immunosuppressant

## Abstract

Macrolides are a diverse class of hydrophobic compounds characterized by a macrocyclic lactone ring and distinguished by variable side chains/groups. Some of the most well characterized macrolides are toxins produced by marine bacteria, sea sponges, and other species. Many marine macrolide toxins act as biomimetic molecules to natural actin-binding proteins, affecting actin polymerization, while other toxins act on different cytoskeletal components. The disruption of natural cytoskeletal processes affects cell motility and cytokinesis, and can result in cellular death. While many macrolides are toxic in nature, others have been shown to display therapeutic properties. Indeed, some of the most well known antibiotic compounds, including erythromycin, are macrolides. In addition to antibiotic properties, macrolides have been shown to display antiviral, antiparasitic, antifungal, and immunosuppressive actions. Here, we review each functional class of macrolides for their common structures, mechanisms of action, pharmacology, and human cellular targets.

## 1. Introduction

The study of macrolides over the past few decades has revealed a varied group of molecules with a range of structures and functions. Macrolides are hydrophobic compounds characterized by a macrocyclic lactone ring that typically contains at least 12 elements, while the remaining structure varies greatly between different classes of molecules [1,2]. 

Many macrolides have been discovered that display pharmaceutical properties, giving them potential as antibiotic, antiviral, antiparasitic, antimycotic, or immunosuppressant drugs [2,3]. While naturally produced macrolides may have limited use as drugs due to their instability in stomach acid, poor pharmacokinetics, and adverse side effects, synthetic macrolide derivatives have been developed to overcome these issues [1]. Many macrolides also exhibit toxic bioactivity, which may be a cause of the adverse side effects observed in response to the administration of macrolide drugs. Indeed, one of the largest classes of macrolides is toxins, many of which are produced from marine sources [4]. On the other hand, some macrolide mechanisms of toxicity have been manipulated for use in therapeutic applications.

The focus of this review was to compare and contrast five major functional classes of macrolides from a variety of natural sources: toxins, antibiotics, antivirals/antiparasitics, antifungals, and immunosuppressants. Each subclass has been reviewed for structural similarities, mechanisms of action, pharmacology, and side effects in human beings. For a comprehensive review of macrolides from marine sources, see the review written by Zhang et al. in 2021 [4].

## 2. Macrolide Classes

### 2.1. Toxins

Macrolide toxins originating from marine and microbial sources represent a vast library of chemical structures with varying mechanisms of toxicity. Marine macrolide toxins are of particular interest, as the number of known toxins has increased greatly over the past 50 years. Increasing knowledge of the biochemistry of these compounds has explained a wide range of toxic effects, from quick paralysis to more gradual changes in target cells. Marine macrolide toxins have also been of interest due to their potential therapeutic properties against cancer and fungal or parasitic infections, and their ability to act as immunosuppressants [3,4]. Toxins produced by bacterial species, such as the mycolactones from *Mycobacterium ulcerans*, also have a wide range of effects on host cells, many of which have yet to be discovered [5].

#### 2.1.1. Structures

The structures of the macrolide toxins reviewed in this paper are variable; however, the majority of the structures consist of two major components: (1) the macrocyclic ring, which can vary in size, conjugated dienes, and functional groups, and (2) the acyclic side chain, which is often referred to as the “tail”, and can also vary in structure and length between different toxins. The major subclasses of macrolide toxins are summarized in Table 1.

#### 2.1.2. Mechanisms of Action

##### Inhibition of Actin Polymerization

Many of the macrolide toxins discussed in this review directly interact with major components of the eukaryotic cell cytoskeleton, including actin, by causing either an inhibition or an increase in polymerization. Cytoskeletal proteins play vital roles in eukaryotic cell health, and disruption of the natural formation of these proteins causes some of the cytotoxic effects observed upon exposure to macrolide toxins [6]. For example, the exchange between G-actin monomers and F-actin polymer filaments is a delicate balance essential to cellular mobility and cytokinesis [6,7]. 

The trisoxazole-ring-containing macrolides, such as the kabiramides, jaspisamides, mycalolides, and ulapualides, are synthesized by marine sponges and bind actin specifically and with high affinity. These toxins cause cellular death by splitting actin filaments (F-actin) and sequestering actin monomers (G-actin) [7,8,9]. Wada et al. studied the interaction of kabiramide D with various intracellular proteins and found that it bound to actin, but not to other proteins it was exposed to, indicating a high specificity for actin [10]. Klenchin et al. performed X-ray structural analyses of kabiramide C and jaspisamide A when bound to actin in order to determine the binding sites of the toxins that lead to actin filament inhibition. Results from these studies suggest that the trisoxazole rings of the toxins interact with G-actin subdomain 1, and the toxins’ aliphatic side chains insert into the hydrophobic cavity between actin subdomains 1 and 3 [7]. Interestingly, this is the same binding site used by the gelsolin family of actin-binding proteins that regulate actin polymerization as part of normal cellular processes [11]. This indicates that (1) actin’s hydrophobic cleft is a highly adaptable binding site that displays interactions with multiple unrelated compounds [11], and (2) some macrolide toxins can be considered biomimetic molecules that directly compete with natural actin-binding proteins to bind at the hydrophobic cleft [7,12]. Indeed, Tanaka et al. performed competition assays by utilizing a fluorescently labeled actin conjugate, the emission spectrum of which shifted upon binding gelsolin or kabiramide C. This work showed that kabiramide C was able to displace the stable bond between gelsolin and G-actin, but gelsolin did not do the same to the kabiramide C–G-actin complex [12].

Many other macrolide toxins interact with actin to impose their cytotoxic effects. Swinholide A, a macrodiolide that also originates from a marine sponge, binds two G-actin monomers to itself, creating a break in filamentous actin strands [12,13,14]. Halichondramide (a trisoxazole) and ankaraholide (a macrodiolide) are also believed to inhibit the polymerization of actin through sequestration of monomeric actin, which caps the barbed end of actin and severs F-actin [14,15]. The latrunculin toxins are macrolides that lack a long side chain [10]. Latrunculin, mycalolide B, and aplyronine toxins bind to G-actin monomers in a 1:1 complex that renders actin unable to polymerize [9,15,16,17]. Reidispongiolides, sphinxolides, aplyronines, mycalolides, ulapualides, halichondramides, and kabiramides all target the barbed end of actin and exhibit cytotoxic properties through their interaction with filamentous actin [18]. 

Structural studies of toxins that have actin filament capping activity from Allingham et al. suggest that the aliphatic tail of macrolide toxins is a major contributor to their high affinity towards actin filaments. In all of the actin–toxin complexes studied, the aliphatic tail was the structural component that made contact with the bottom portion of the hydrophobic cleft within actin. The macrocyclic ring formed either a tight or loose interaction with a hydrophobic area on the side of actin, depending on the structure of the macrocyclic ring, with the first nine atoms of the ring consistently interacting and the variable regions of the ring interacting with different degrees of affinity [18]. This structural study gives insight into the importance of the macrocyclic ring in toxin affinity towards actin, and could help explain why dissimilar macrolide toxins all bind to actin at the same binding site.

##### Increased Actin Polymerization

Most of the actin-interacting toxins discussed thus far prevent the polymerization of actin by different mechanisms, but actin polymerization in excess is another cause of cytotoxicity. Actin polymerization is vital to cellular processes including motility, cellular division, and cell morphology [15,16,19]. A disruption in these processes can be catastrophic for cell vitality. Mizuno et al. studied the effects of goniodomin A, a polyether macrolide isolated from the dinoflagellate *Goniodoma pseudogoniaulax*, on cultured 1321N1 human astrocytoma cells. It was found that exposure to goniodomin A induced a long needle-like structure to grow from the cells. Goniodomin A also caused the exposed cells to make more F-actin in a concentration- and time-dependent manner [20]. The jasplakinolides have been shown to induce actin polymerization in vitro and in vivo. One of the ways in which jasplakinolides induce this effect is by increasing the on-rate of actin subunits nearly two-fold [15].

##### Modulation of Actomyosin ATPase Activity

Interference with actomyosin ATPase activity, which drives muscle contraction, is another mechanism of macrolide toxicity [21]. Goniodomin A has been reported to be highly ichthyotoxic and capable of modulating actomyosin ATPase activity [21,22]. Goniodomin A decreased rabbit skeletal actomyosin ATPase activity due to an alteration in the conformation of actin upon exposure to goniodomin A. This effect was determined to be dependent on the concentration of actin, but not the concentration of myosin, further supporting the idea that goniodomin A affects actin’s ability to participate in actomyosin ATPase activity [21].

##### Prevention of Tubulin Polymerization

Tubulin is another cytoskeletal protein that can be targeted by macrolide toxins and is critical to cell division, since it forms the mitotic spindle. Aplyronine A is capable of binding to tubulin in association with actin [17,23]. Spongistatin 1 (also known as altohyrtin A) is a cytotoxic macrolide that has been found to inhibit tubulin polymerization and arrest mitosis [24]. Further studies of spongistatin 1 have found that it competitively inhibits GTP exchange and the formation of Cys-12—Cys-201/211 cross links on tubulin [25]. Halichondrin B, a polyether macrolide isolated from *Halichondria okadai*, has a unique mechanism of action compared to other tubulin inhibitors. Halichondrin B has been shown to inhibit the growth, but not the shortening, of microtubules. It also sequesters tubulin subunits into nonfunctional aggregates, which arrests mitosis at the G2–M phase and results in apoptosis [26,27]. 

##### Wiskott Aldrich Syndrome Proteins (WASPs)

The Wiskott Aldrich syndrome proteins (WASPs) are a family of scaffold proteins that transduce signals for the dynamic remodeling of the actin cytoskeleton, which they do through the interaction of their C-terminal verprolin-cofilin-acidic (VCA) domain with actin-related protein 2/3 (ARP 2/3) actin nucleating complex. WASPs (expressed by hematopoietic cells) and N-WASPs (more widely expressed) self-regulate by auto-inhibition when VCA is sequestered from ARP 2/3, and begin actin polymerization upon GTPase binding, which results in a conformational change allowing VCA to release and bind ARP 2/3. Mycolactone, a polyketide toxin secreted by *Mycobacterium ulcerans*, mimics the endogenous GTPase CDC42, leading to an interference of WASP auto-inhibition and uncontrolled ARP 2/3 activation [5,28]. In this way, mycolactone has an indirect effect on actin polymerization, while the other toxins discussed exert a direct effect on actin and other microtubule components.

##### Host Immunomodulation

Mycolactone is the causative agent of the necrotizing disease Buruli ulcer (BU) [5,28,29]. A hallmark of BU is a poor inflammatory reaction, which is believed to be a result of mycolactone’s immunosuppressive nature. Mycolactone has been shown to significantly inhibit the production of interleukin-2 (IL-2) from T cells and also to inhibit tumor necrosis factor (TNF)-dependent activation of NF-κB, a key immune transcription factor, in T cells [30]. TNF production was shown to be greatly reduced in human peripheral blood monocytes when exposed to pathogenic *M. ulcerans* extracts and lipopolysaccharide (LPS), demonstrating the strong immunosuppressive action of mycolactone and its ability to suppress the inflammatory response typical of LPS exposure [30,31]. Coutanceau et al. studied the effects of mycolactone on mouse and human dendritic cells, which typically initiate and regulate immune responses. In both species, exposure to noncytotoxic concentrations (<50 ng/mL) of mycolactone inhibited the maturation of dendritic cells both phenotypically and functionally, an effect that was not reversed upon removal of mycolactone. In human peripheral blood derived dendritic cells, mycolactone had a selective effect on the production of cytokines; IL12, TNFα, and IL-6 were only marginally affected, while a near elimination of the chemokines macrophage inflammatory protein (MIP) 1α, MIP-1β, interferon γ–inducible protein 10, and monocyte chemoattractant protein 1 was observed upon exposure to just nanomolar concentrations of mycolactone [29].

##### Prevention of Protein Secretion

Sec61 is a translocon that allows for the transport of almost all secretory proteins in eukaryotic cells into the ER lumen [32]. Apratoxin A, discovered in the marine cyanobacterium *Moorea bouillonii*, directly binds to the Sec61α central subunit to prevent protein translocation into the ER. Paatero et al. showed that apratoxin A blocked ER translocation of preprolactin, binding immunoglobulin protein (BiP), human hepatocyte growth factor (HGF), and other proteins with similar potency. This suggests that apratoxin A does not display substrate-selective inhibition of ER translocation [33]. Mycolactone also interacts with Sec61 to induce a conformational change in the pore-forming subunit Sec61α, which prevents protein translocation and causes proteins to break down via the ubiquitin-proteasome system in the cytosol [5,34]. Further studies have shown that mycolactone directly targets the α subunit of Sec61 to block the production of integral membrane proteins [35]. Expression of mutant and mycolactone-resistant Sec61α in mycolactone-treated T cells revived their homing potential and effector functions, and, when expressed in macrophages, restored IF-γ receptor-mediated antimicrobial responses [35]. This suggests that the macrolide toxin mycolactone is a mediator of immunomodulatory effects through the Sec61 translocon.

##### mTOR Downregulation

The mechanistic Target of Rapamycin (mTOR) is a serine/threonine protein kinase that is involved with cell survival, proliferation, transcription, and translation. mTOR interfaces with various proteins to form two distinct complexes with different cellular roles: mTORC1 and mTORC2 [36]. Bieri et al. studied the apoptotic effects of mycolactone on the mTORC1-targeted ribosomal protein S6 and the mTORC2-targeted protein kinase Akt in L929 fibroblasts. Mycolactone prevented the phosphorylation of Akt, which led to the dephosphorylation and activation of the Akt-targeted transcription factor FoxO3. Activation and upregulation of the FoxO3 target gene BCL2L111 (Bim) drives the expression of the pro-apoptotic regulator Bim, which leads to apoptosis in mammalian cells [36]. Bae et al. showed that halichondramide also interacts with the Akt/mTOR signaling pathway in A549 lung carcinoma cells. Halichondramide suppressed Akt signaling, which downregulated the activation of mTOR and its downstream effectors p70 S6 kinase and eukaryotic initiation factor 4E-binding protein 1. Since the Akt/mTOR signaling pathway is often found to be highly active in cancer cells, this could be a potential route of therapy for cancer [37].

#### 2.1.3. Human Health Applications

While the macrolides discussed in this section have all been discovered due to their toxicity, some of them have been shown to display anticancer, antibiotic, and/or antifungal properties when used in the context of human health. Spongistatin 1, a natural marine product that has a cytotoxic microtubule-targeting mechanism of action, has also been shown to be a potential anticancer agent. Xu et al. studied the effect of (+)-spongistatin 1 on a wide range of human cancer cells and nonproliferating normal human fibroblast cells. It was found that (+)-spongistatin 1 displayed subnanomolar growth inhibition on all of the cancer lines tested, with an IC50 range between 0.037–0.5 nM. In the same concentration range, (+)-spongistatin 1 displayed no cytotoxicity against the quiescent human fibroblast cell line, indicating its potential as a specific anticancer therapeutic [25]. The actin-targeting macrolide latrunculin has also been shown to have anticancer properties against peritoneal dissemination of gastric cancer. Latrunculin A induced apoptosis in MKN45 and NUGC-4 cell lines by activating the caspase-3/7 pathway [38]. Halichondramide, another actin-targeting toxin, displays antiproliferative and antimetastatic activity against PC3 human prostate cancer cells. Shin et al. showed that halichondramide suppressed the expression of phosphatase of regenerating liver-3 (PRL-3), which tends to be overexpressed in metastatic cells, and downregulated mRNA expression of genes associated with metastasis, including matrix metalloproteinase-2 (MMP2), MMP9, and N-cadherin in PC3 cells [39]. Eribulin mesylate, a synthetic derivative of the microtubule growth inhibitor halichondrin B, was the subject of successfully completed clinical trials and has been approved by the FDA for the treatment of metastatic breast cancer and liposarcoma [40]. Eribulin has been shown to be significantly more antiproliferative against breast cancer cell lines that demonstrate higher βIII-tubulin expression. The βIII-tubulin isotype tends to be overexpressed in cells that have developed resistance to other microtubule-inhibiting drugs, which makes eribulin a potentially effective treatment option in these cases [41].

Many macrolide toxins have been investigated for their antimicrobial properties. Halichondramide has shown strong antifungal activities against *Candida albicans* and *Trichophyton mentagrophytes* [42]. Venturicidin A, produced by actinomycetes, has been shown to be an effective adjuvant in the treatment of drug-resistant bacterial infections. When used in combination with the antibiotic gentamicin, venturicidin A increased bactericidal activities against multidrug-resistant *Staphylococcus*, *Enterococcus*, *Pseudomonas aeruginosa*, and methicillin-resistant *Staphylococcus aureus* (MRSA). The adjuvant mechanism of action is speculated to be related to the blocking of proton flow through ATP synthase by venturicidin A, resulting in a higher concentration of extracellular protons and increase in bacterial gentamicin uptake [43]. Tolytoxin, produced by the cyanobacterium *Scytonema ocellatum*, has been shown to display antifungal activity. Patterson and Bolis showed that exposure of *S. ocellatum* to fungal cell wall polysaccharide homogenates markedly increased the production of tolytoxin, especially when in the presence of fungal chitin and carboxymethylcellulose, indicating tolytoxin is an inducible protective agent used by *S. ocellatum* to survive fungal infections [44].

### 2.2. Antibiotics

Since the so-called “golden era” of antibiotic discovery between the 1930s and 1960s, macrolide antibiotics have been widely studied and prescribed for the treatment of infectious disease [45]. While antibiotics are used as first-line agents in treating infectious disease driven by bacteria, macrolide antibiotics often also exert immunomodulatory effects. In addition, recent studies have revealed potential clinical benefits of macrolides in the treatment of chronic inflammatory airway diseases [46]. Macrolide antibiotics display bacteriostatic and bactericide activity against various Gram-positive and Gram-negative species, as well as some Gram-indeterminate bacteria [45,47,48]. Because of their low toxicity, macrolide antibiotics are often selected as the safest option for antibacterial treatment [47]. This advantage is enhanced as allergic reactions to the macrolide antibiotics are noted to be rare; however, there have been some cases reported in the literature [49,50,51,52].

#### 2.2.1. Structures

Macrolide antibiotics are typically 12- to 16-membered macrolactone rings that contain various amino sugars and lack the acyclic side chain characteristic of macrolide toxins (Table 2) [4,47,53,54]. For example, erythromycin, the first macrolide antibiotic to be discovered, is a 14-membered macrolide that has a wide antimicrobial spectrum [47]. Commonly prescribed macrolide antibiotics include azithromycin, erythromycin, and clarithromycin. These macrolides consist of a 14- or 15-membered alkylated lactone ring with hydroxyl groups on C3, C5, C6, C11, and C12 and a desosamine and decladinose sugar on C3 and C5 [4,47,55]. 

#### 2.2.2. Mechanisms of Action

##### Inhibition of Bacterial Protein Synthesis

One of the major mechanisms of macrolide antibacterial action is the interference with protein synthesis within bacterial cells [56,57,58,59]. The macrolide antibiotics all display similar binding to the large (50S) ribosomal subunit in bacteria [47,48,53,58,59,60]. Studies have suggested that specific binding is to the 23S ribosomal RNA molecule within the 50S ribosomal subunit [47,48,53]. The lactone ring of the macrolide has been shown to bind to the wall of the nascent peptide exit tunnel of the ribosome through hydrophobic interactions involving nucleotides A2058 and A2059 in *E. coli* [53,54]. The exit tunnel is an essential structural component of the ribosome through which newly synthesized proteins are released. When macrolide antibiotics bind to the exit tunnel, the release of nascent peptides and thus translation are inhibited. As protein inhibition continues, free tRNAs from the cell are used up, and translation is eventually halted [54]. Although macrolide antibiotics all bind to the same ribosomal site, there are differences in their mechanisms of action. For instance, macrolides with a shorter C5-side chain do not disrupt peptide bond formation, but rather inhibit the elongation of longer peptides through steric hindrance. Longer C5-side chains can span into the peptidyl transferase center to directly stop peptide bond formation [53,54]. 

While it is generally accepted that macrolide antibiotics target bacterial translation by binding to the nascent peptide exit tunnel of the bacterial ribosome, this classic model of macrolide action has been suggested to be more complex than originally proposed [59]. Studies have shown that macrolide antibiotics allow the exit of some oligopeptides in a context-specific manner, while inhibiting the synthesis of specific proteins [61,62]. This contradicts the idea that translation is completely halted. Vazquez-Laslop and Mankin performed ribosome profiling experiments and determined that when a macrolide antibiotic binds to a ribosome in the process of translating mRNA, the ribosome will stall translation at specific sequence sites called macrolide arrest motifs. The exact mechanism of translational halting has yet to be elucidated, but it likely has to do with the macrolide binding the peptidyl transferase center, which interferes with peptide bond formation between specific amino acids with certain charges or side chain configurations [59]. 

##### Impairment of Biofilm Synthesis

Infection with certain bacteria, such as *Pseudomonas aeruginosa* in the lungs, can lead to biofilm formation in the small airways and exacerbation of infection [63,64]. A biofilm consists of a collection of bacteria embedded in a polysaccharide matrix, which can adhere to itself and to the airway mucosa. This matrix allows bacteria to evade phagocytosis, most antibiotics, and the ciliary forces of the epithelial cells in the host airway [64,65]. Macrolide antibiotics, including erythromycin and clarithromycin, have been effective in the treatment of biofilm-producing bacterial infections. The major mechanisms of biofilm impairment by macrolide antibiotics are thought to be (1) reduction of mucus secretion by human airway cells via blockage of chloride ion transport and thus osmosis towards the airway lumen; and (2) inhibition of glycocalyx production by bacterial cells [64]. Interestingly, the number of members in the lactone ring could have an effect on the macrolide’s ability to inhibit biofilm formation. Yanagihara et al. found that the 14-membered macrolide clarithromycin was more effective against *Pseudomonas aeruginosa* biofilm production than the 16-membered josamycin. It is unknown whether this is true for all 14- and 16-membered macrolide antibiotics [66].

#### 2.2.3. Pharmacology

Macrolides have been preferentially selected as antibiotics due to their enhanced tissue penetration and inhibition of many common pathogens [67,68]. When considering the use of antimicrobials in a clinical setting, three major pharmacokinetic and pharmacodynamic parameters are generally considered: (1) the peak plasma concentration of the antibiotic; (2) the area under the plasma concentration–time curve; and (3) the minimum inhibitory concentration [68]. Taking these parameters into consideration helps to ensure effective and safe treatment strategies. Most macrolide antibiotics need to be administered multiple times per day in order to avoid a high peak serum concentration which could lead to side effects. The timing of each dose is dependent on the half-life of the specific macrolide [68]. 

Once in the human body, macrolide antibiotics differ in pharmacokinetics based on their structure. For example, erythromycin is sensitive to acid and therefore degrades in the stomach. Clarithromycin and azithromycin are both acid-stable, leading to less degradation in the stomach, a longer half-life, and higher tissue concentrations [69]. In the bloodstream, macrolides bind preferentially to the alpha-1-acid glycoprotein (AGP), a binding protein that is second highest in concentration after albumin [68,69,70]. Binding to AGP is variable; erythromycin tends to be 70–80% bound to AGP in plasma [70], whereas azithromycin can be found up to 93% unbound in plasma [71]. In the intestine, macrolide absorption is thought to be limited by the P-glycoprotein ABCB1 efflux transporter [72,73]. Macrolide antibiotics are taken up and excreted by bile (approximately 60–70%) through a mechanism that has yet to be discovered [56,74]. The macrolide antibiotics azithromycin, erythromycin, and clarithromycin bind to bile acids with varying affinities, but appear to bind stronger with greater hydrophobicity [56]. These interactions can guide the selection of antibiotics depending on the length of treatment necessary. For instance, reduced binding of the antibiotic to bile will reduce the excretion rate of that antibiotic, suggesting the use of less hydrophobic antibiotics for long-term treatment. Choosing a more hydrophobic antibiotic will offer stronger binding to bile acids and therefore faster transport from the body [56].

The macrolide antibiotics also have pharmacokinetic dynamics once inside target bacterial cells. As discussed previously, macrolides bind the nascent peptide exit tunnel of the large ribosomal subunit in bacterial cells. The dissociation rate of the macrolide from the ribosome and the peptidyl-tRNA drop-off rate have an effect on the ability of the macrolide to inhibit translation. For example, bacterial protein translation can continue if the macrolide drug dissociates and amino acids are added to the growing polypeptide before another antibiotic molecule can bind [54]. Macrolide antibiotics also compete with other macrolides to bind the bacterial ribosome. Most macrolide antibiotics have dissociation constants in the 10^−8^ to 10^−7^ M range [48].

#### 2.2.4. Side Effects and Human Targets

##### Gastrointestinal Motility

One of the most common side effects of macrolide antibiotics is gastrointestinal distress, characterized by diarrhea, abdominal pain, nausea, and vomiting [75,76]. Erythromycin and its derivatives induce phase-III-like contractions in the human gastrointestinal tract during the interdigestive state that are similar to the contractions induced by motilin, a peptide that stimulates motor activity in human smooth muscle receptors [77]. Satoh et al. reported that the erythromycin derivative EM574 directly stimulates smooth muscle contraction by acting on motilin receptors in the human gastric antrum in vitro [77]. Other in vitro studies have suggested that erythromycin A mimics motilin, which can cause gastrointestinal distress [78]. Azithromycin has also been found to promote gastrointestinal motility, and can be used as a therapeutic to treat gastroparesis [79]. Itoh et al. studied a few different macrolide antibiotics and their motor-stimulating abilities in the canine digestive system. It was found that the 14-membered macrolides erythromycin and oleandomycin had strong stimulatory effects on gastrointestinal motor activity, while the 16-membered macrolides leucomycin, acetylspiramycin, and tylosin did not elicit the same response. These findings point to a structure–function relationship between the macrolide antibiotics and gastrointestinal side effects [80]. 

##### Electrophysiological Effects on Heart

Electrophysiological effects on the heart are possible when taking macrolide antibiotics. These effects include prolonged cardiac repolarization [81], a proarrhythmogenic effect [82,83], and potentially fatal ventricular tachycardia [84]). Erythromycin has been reported to be associated with doubled risk of sudden cardiac death, and nearly five-fold increased risk of cardiac death in patients concurrently taking drugs that inhibit cytochrome P-450 3A [83]. This is likely due to the fact that erythromycin is extensively metabolized by the cytochrome P-450 3A isozyme [83]. However, the cardiovascular risks associated with the use of these macrolide antibiotics may be reduced with other macrolide antibiotics such as azithromycin [85,86,87]. 

##### Cholestatic Hepatitis

Cholestatic hepatitis is a rare but well documented side effect of macrolide antibiotic use. A cholestatic state can cause vomiting, transaminase elevation, and increased bilirubin, amongst other symptoms. Cholestasis is thought to be caused by a hypersensitivity to the macrolide drug, and not from a state of toxicity [88,89,90]. 

##### Immunomodulation

In addition to their antibacterial properties, macrolide antibiotics have been studied for their anti-inflammatory and immunomodulatory properties. Many cells involved in the immune response are influenced in some way by macrolide antibiotics [65]. Erythromycin prevents the release of IL-8, epithelial cell derived neutrophil attractant (ENA-78), and MIP-1 from macrophages and leukocytes [3,91]. There is evidence to suggest that 14- and 15-membered macrolide antibiotics, but not 16-membered macrolides, decrease the hypersecretion of proinflammatory cytokines and chemokines in cell cultures, animal models, and people with chronic inflammatory pulmonary disease, which appear to be mediated through inhibition of extracellular signal-regulated kinase (ERK) 1/2 or downstream transcription factors [3]. 

Macrolide antibiotics have been shown to interact with the chemotaxis of neutrophils. In patients with chronic inflammatory pulmonary diseases, inflammatory signals cause neutrophils and other inflammatory cells to migrate to the airway, where they require adhesion molecules to stay in place [3]. It has been reported that the long-term use of erythromycin in diffuse panbronchiolitis significantly reduces the number of neutrophils and the neutrophil chemotactic activity in bronchoalveolar fluid [92,93]. This is understood to be due to the reduced expression of adhesion molecules and decreased production of chemoattractants [3,94,95]. Erythromycin has also been reported to downregulate the integrin CD11b/CD18 [96]. Roxithromycin therapy can cause a significant decrease in the expression of Mac-1 (CD11b/CD18) expression on peripheral neutrophils from patients with diffuse panbronchiolitis [97]. This decrease in Mac-1 expression was associated with reduced neutrophil numbers in bronchoalveolar fluid [97]. Not only do macrolides alter neutrophil release, but macrolides also likely alter eosinophil recruitment through inhibition of the secretion of eosinophil-chemotactic cytokines, including chemokine ligand 5 (CCL5) and eotaxin [98]. The immunomodulatory side effects of macrolides have allowed them to be used to treat chronic inflammatory disorders characterized by neutrophil dominance, such as panbronchiolitis, rhinosinusitis, and cystic fibrosis [3].

### 2.3. Antivirals and Antiparasitics

The discovery of macrolides that display antiviral and anthelmintic properties has greatly reduced the incidence of viral and parasitic diseases. Avermectins were discovered to be produced by *Streptomyces avermitilis* in 1973, and display highly specific anthelminthic, but generally not antibacterial or antifungal, activity [99,100]. Ivermectin is the most widely used avermectin derivative due to its safety and efficacy. After being used successfully in the agricultural and veterinary fields, ivermectin was introduced to treat onchocerciasis (also known as river blindness) in resource-poor tropical populations in 1987, where it was given to patients free of cost. This effort has been largely successful in the elimination of onchocerciasis in those regions, and ivermectin has been deemed a “wonder drug” [100,101]. Since then, ivermectin has been shown to have broad-spectrum antiparasitic properties, and has been used to treat lymphatic filariasis, strongyloidiasis, scabies, and head lice [100,102]. Balticolid, a plecomacrolide produced by a marine-derived ascomycetous fungus, was discovered more recently in driftwood off the Baltic Sea in Germany, and has been shown to display anti-HIV and anti-herpes simplex type I activity [103,104,105]. The bryostatins are a class of highly oxygenated macrocyclic lactones originating from marine bryozoan *Bugula neritina* [106,107]. There have been over 20 bryostatins isolated to date, some of which display antiviral activity [107].

#### 2.3.1. Structures

Ivermectin, one of the most widely used antiparasitic macrolides, is a semisynthetic mixture of two avermectin derivatives, comprising 80% 22, 23-dihydroavermectin B1a and 20% 22, 23-dihydroavermectin B1b [100,102,108]. Balticolid is a 12-membered macrolide of the plecomacrolide class [103]. A summary of relevant antiviral and antiparasitic macrolides is presented in Table 3.

#### 2.3.2. Mechanism of Action

##### Modulation of Parasite Ion Channels and Receptors

Both components of ivermectin are hydrophobic molecules that bind various Cys-loop receptors present in invertebrates, including glutamate-gated chloride channels (GluCl), histamine-gated chloride channels, glycine receptors, the α-aminobutyric acid receptor (GABA_A_R), and the α-7-nicotinic acetylcholine receptor (cAChR) [109,110,111]. In the case of GluCl receptors, which are the most well characterized to date, ivermectin binds the transmembrane domains of the receptor at the extracellular surface of the cell membrane, leading to opening and stabilization of the pore [109]. Althoff et al. showed that upon binding to ivermectin, a rotational shift of the transmembrane domains towards the extracellular surface occurs, which expands the pore size [112]. This causes the hyperpolarization of parasitic neuronal and muscular cells and the blocking of inhibitory neurotransmitters, which leads to parasite paralysis and death [109,113]. 

##### Inhibition of Nuclear Transport

While ivermectin is primarily used for its broad-spectrum antiparasitic activity, it has also been shown to have antiviral properties [102,113]. The proposed mechanism of ivermectin action is the inhibition of nuclear transport, which is mediated by the importin α/β1 heterodimer. Without the translocation of viral species proteins (including HIV-1 and SV40), viruses cannot replicate [113,114,115]. This mechanism seems to affect multiple RNA viruses, including Dengue virus 1–4 [116], West Nile virus [117], Venezuelan equine encephalitis virus [118], and influenza [113,119]. Ivermectin has recently been shown to inhibit the replication of SARS-CoV-2 in vitro [113,120,121]. Since SARS-CoV-2 is an RNA virus, one can conclude that this is a result of the same nuclear transport halting mechanism; however, the definitive mechanism of action against SARS-CoV-2 has yet to be determined. In a recent pilot clinical trial, the antiviral effects of ivermectin were studied in 30 patients with mild PCR-confirmed COVID-19. Patients were randomized into groups that received either the standard-of-care treatment, or the standard plus a specific dose of ivermectin. The use of ivermectin in conjunction with the standard care of treatment resulted in decreased viral load, reduced symptoms, and a reduction in the time to obtain two consecutive negative SARS-CoV-2 PCR tests, which occurred in a dose-dependent manner. This study highlights the potential of ivermectin as a safe adjuvant in the treatment of COVID-19 [121]. 

##### Protein Kinase C Modulation

Several bryostatins have been reported to be antineoplastic agents [106,122]; however, recent research has shown bryostatin to be a successful treatment for Chikungunya virus [123]. Bryostatin binds to and affects the activity of various protein kinase C (PKC) isoforms, which are essential signal transducers. The result of exposure to bryostatin 1 is autophosphorylation, protein translocation, and ubiquitination of PKC. Bryostatin 1 can also cause cell death by affecting regulatory proteins related to the cell cycle [107]. Endogenous regulation of PKC occurs through the binding of diacylglycerol (DAG), which binds to C1 domains of PKC [123]. The C ring of bryostatin has been shown to interact with PKC-C1-binding domains at concentrations that are orders of magnitude lower than for DAG. Recent findings indicate that there are additional unidentified pathways for Chikungunya virus intervention by bryostatins [123].

##### Reactivation of Latent HIV

Bryostatin-1 is considered to be one of the most promising anti-AIDS drugs. AIDS has been difficult to fully cure due to the presence of latent HIV that stores replication-competent provirus [107,124]. After antiviral treatment is complete, patients often experience a rebound effect, which is caused by latent HIV beginning to replicate [120]. Bryostatin-1 reactivates latent HIV, which can then be followed by antiretroviral treatment to completely eradicate the virus [107,124]. It has been proposed that bryostatin-1 reactivates latent HIV through the activation of PKC, which has been shown to activate transcription of HIV through NF-κB [125]. Mehla et al. sought to determine the specific PKC isoforms involved in the reactivation of HIV by bryostatin-1 by utilizing specific PKC inhibitors on latently infected THP-p89 cells. The novel PKC inhibitor rottlerin, which inhibits PKC-δ and -θ, was shown to halt bryostatin-1-induced HIV reactivation in a dose-dependent manner, implying PKC-δ and/or -θ isoforms are involved in the pathway [126]. Other latent reversal agents are unable to effectively reactivate latent HIV within the central nervous system, whereas bryostatin-1 is able to permeate the blood–brain barrier, making it advantageous compared to other latent reversal agents for HIV treatment [107,125]. A phase I clinical trial of bryostatin-1 use in HIV-positive patients found the drug to be safe at the doses administered, with mild side effects observed in only 2 out of 12 patients [127].

#### 2.3.3. Pharmacology

Ivermectin has displayed high potency when administered orally in humans. To treat onchocerciasis, the optimal dose of ivermectin is 150 μg/kg administered one to three times per year [102,128,129,130]. The treatment of scabies has been successful at doses of 200 μg/kg, often in two or three repeats. After oral administration of ivermectin, both healthy and onchocerciasis patients showed a decrease and then second rise in plasma concentrations of the drug, which suggests recycling via the enterohepatic mechanism [102,121]. Since ivermectin is lipophilic, it shows a wide distribution throughout the body [102]. It is up to 93% bound to plasma proteins, with a specific binding site on albumin displaying an association constant of 2 × 10^8^ mol^−1^ [102,130]. Ivermectin is metabolized in the liver by cytochrome CYP1A and CYP3A4 complexes, where at least 10 metabolites are produced [102,121]. Excretion occurs almost exclusively through the feces, with only 1% through urine [102].

Bryostatin-1 is administered through an intravenous injection or by continuous infusion. The circulating concentration of bryostatin-1 in humans is very low, which makes it difficult to detect by mass spectrometry or other methods. Studies in mice have shown bryostatin-1 to accumulate in the lungs, liver, gastrointestinal tract, bone marrow, and fat after injection. After an intravenous injection of bryostatin-1, levels in the brain were seen to briefly spike in mice. This further supports the notion that bryostatin-1 can cross the blood–brain barrier [131,132]. In mice, the excretion pathway seems to be primarily through the urine, with about 23% of bryostatin-1 being excreted within 12 h after intravenous administration. Following intraperitoneal injection, excretion is more evenly balanced between urine and fecal routes [132].

#### 2.3.4. Side Effects and Human Targets

The side effects of treatment with ivermectin and bryostatin-1 have been reported to be generally low. Ivermectin is considered to be safer and more effective than the alternatives, but some moderate side effects to treatment can include rashes, skin edema, bone pain, enlargement of lymph nodes, headaches, and fevers [121,129,133]. Bryostatin-1 can have an adverse effect in the treatment of HIV, since it stimulates the expression of several silent genes in unrelated cells, including the expression of inflammatory factors, but this can be controlled by pairing it with other latent reversal agents to mediate side effects [107]. 

Both ivermectin and bryostatin-1 have been found to have human cellular targets that make them attractive candidates for the treatment of cancers and neurological disorders. Ivermectin has proven efficacy against many cancer types, including leukemia, ovarian cancer, breast cancer, pancreatic cancer, glioblastoma, and more. Some of its effects on cancerous cells are the inhibition of cell proliferation, induction of apoptosis, autophagy, and reversion of tamoxifen resistance [107,121,134,135]. 

Bryostatin-1 has been investigated for the treatment of cancer and neurological issues, in addition to its known efficacy in HIV treatment [107,123,125]. Broystatin-1 has been found to increase the sensitivity of human glioma cells to radiation therapy, improving outcomes of treatment [136]. Since bryostatin-1 is an activator of PKC, it has been shown to increase the expression of brain-derived neurotrophic factor in the hippocampus, which can lead to improved memory and lessened feelings of depression [107,137]. In treatment and prevention of Alzheimer’s disease (AD), Nelson et al. observed that bryostatin-1 crosses the blood–brain barrier to activate protein kinase C-ϵ in the brain, which is involved with learning and memory functions, and could potentially prevent or reverse the synaptic loss in patients with AD [138]. Bryostatin-1 has also been shown to effectively heal blood–brain barrier injuries through the upregulation of tight junction proteins and the modulation of PKC [139].

### 2.4. Antifungals

Macrolide antibiotics that target fungi, including amphotericin B and nystatin, amongst others, are some of the most effective therapeutics against potentially life-threatening fungal infections in humans [140]. In 1950, nystatin was the first polyene macrolide antifungal compound to be discovered by Rachel Fuller Brown and Elizabeth Lee Haze. It was also the first widely available and effective antifungal treatment. Brown and Haze cultured *Streptomyces noursei* from soil found near a dairy farm in Fauquier County, Virginia, and determined its antifungal properties by screening it against *C. albicans* and *C. neoformans*. Nystatin was then purified and characterized from the *Streptomyces noursei* cultures [141,142]. This work led to the continuation of mycological studies and the subsequent discovery of amphotericin B (AmB) in 1955 at the E.R. Squibb Institute [142,143]. Amphotericin B, which is the current “gold standard” of treatment for fungal infections, was isolated from cultures of *Streptomyces nodosus* found in soil near the Orinoco River of Venezuela and has been shown to have broad antifungal activity, as well as antiparasitic activity [143]. Two additional antifungal compounds were discovered in 1955: natamycin (previously called pimaricin) and filipin [144,145]. Natamycin was isolated from a culture of *Streptomyces natalensis* found in soil from Natal, South Africa [144]. Filipin was named after its place of discovery, the Philippines, where it was isolated from *Streptomyces filipinesis* [145]. Cruentaren A and B were identified more recently and were isolated from *Byssovorax cruenta*. Cruentaren A is most commonly used as an antifungal treatment, as cruentaren B displays a lack of antifungal activity [146,147].

#### 2.4.1. Structures

The majority of the antifungal compounds described in this manuscript are polyene macrolides, while just one (cruentaren) is a salicylate macrolide. Polyenes display a characteristic hydrocarbon chain (or chains) with alternating single and double carbon–carbon bonds, while salicylates are derivatives of salicylic acid. Table 4 provides an overview of macrolide antifungal compounds of relevance.

#### 2.4.2. Mechanism of Action

##### Ergosterol Binding

The polyene macrolide antifungal compounds of interest in this review are ionophores that bind ergosterol, an abundant sterol component of the fungal cell membrane. Binding to ergosterol is one of the most widely accepted mechanisms of action for the polyene macrolides [148,149]. The polyene structures of these macrolides allow them to form complexes with ergosterol, which subsequently cause pore formation in the fungal cell membrane [143]. In 1997, Baginski et al. modeled the interaction between AmB and ergosterol-containing membranes. This work suggested that a transmembrane pore is formed between 7–8 AmB molecules and 7–8 ergosterol molecules, with the polar portion of AmB facing towards the center of the channel and intermolecular hydrogen bonds between adjacent AmB molecules stabilizing the pore [150]. Indeed, it has been shown that AmB binds poorly to phospholipid vesicles without ergosterol, but when ergosterol is present, AmB binds and channels form in the membrane. The model sterol auxotroph *S. cerevisiae*, which requires dietary sterol, was studied as a way to determine the effects of sterol content on polyene antifungal action. It was shown that when sterol content was low, nystatin’s ability to cause K+ leakage and cell death was decreased [151,152]. The transmembrane pores formed by the polyene-ergosterol complexes cause the loss of ions and other small molecules from the fungal cell, resulting in cell death [148,153]. Interestingly, while natamycin is a polyene that binds to ergosterol, it does not permeabilize the fungal cell membrane. The exact mechanism remains unknown, but since natamycin binds strongly to ergosterol, it is thought to be related to the disruption of ergosterol functioning. Natamycin may inhibit the important ergosterol roles of aiding in endocytosis, exocytosis, or vacuolar fusion, leading to fungal cell death [149]. Filipin is another polyene that displays a slightly different mechanism of action: it causes membrane fragmentation instead of pore formation [154].

##### Oxidative Damage

Alternative mechanisms of action, likely to be secondary to the major effects of ergosterol binding, have also been proposed for the polyene antifungals. It has been suggested that AmB invokes oxidative damage. Work by Sokol-Anderson et al. demonstrated that *C. albicans* protoplasts grown in hypoxic conditions showed a reduction in lysis when exposed to AmB when compared to protoplasts grown in normal conditions, suggesting that oxygen is necessary for AmB to damage fungal cells. Results from these experiments also showed that K+ leakage occurred in both conditions, suggesting that AmB could still bind ergosterol [155]. Mesa-Arango et al. showed that AmB induces the production of reactive oxygen species (ROS) by 44 different pathogenic yeast species. In normal conditions, ROS are a byproduct of cellular respiration by the mitochondria. In abnormal conditions that cause intracellular ROS to increase, macromolecules are altered and the cell eventually dies [156]. The addition of catalase, a free radical scavenger, has been shown to protect *C. albicans* protoplasts from the deleterious effects of AmB, indicating that the production of intracellular ROS plays a role in AmB’s ability to kill fungal cells [155]. 

##### Inhibition of ATP Hydrolysis

Cruentaren A, a derivative of salicylic acid, has a different structure than the polyene macrolides, and thus a different mechanism of action. This compound is able to inhibit the growth of several different yeasts, filamentous fungi, and some Gram-positive bacteria [147]. Kunze et al. found that cruentaren A was more active against *S. cerevisiae* when grown on glucose-free media, which led them to hypothesize that cruentaren A could act on a target linked to the mitochondria. The group went on to show that 0.1 μM and 1.0 μM cruentaren A inhibited mitochondrial ATPase hydrolysis by 80–90% in submitochondrial particles of *S. cerevisiae* and beef heart. Based on these findings, it was speculated that cruentaren A interferes with the proton-pumping F0F1-ATPase of the inner mitochondrial membrane [147]. Further work from Kunze et al. looked into the dose-dependent response of F0F1-ATPase activity to cruentaren A, and showed that activity was inhibited half-maximally at 13 and 30 nM concentrations of cruentaren A. By testing samples enriched in F1-ATPase, the group concluded that cruentaren A targets the catalytic F1 domain, not the membrane-bound F0 domain [157]. More research needs to be done in order to elucidate the binding site of cruentaren A on mitochondrial F1-ATPases.

#### 2.4.3. Pharmacology

It has been shown that amphotericin B administered orally results in poor gastrointestinal uptake; thus, intravenous infusion is the preferred route. The behavior of the drug in the body depends on the formulation of amphotericin B [143]. The conventional sodium deoxycholate micellar formulation of amphotericin B (D-AmB) is separated from the deoxycholate upon entering the blood stream, and quickly binds 90–95% to serum proteins, especially β-lipoproteins [143]. The recommended dosage of deoxycholate AmB is 0.75 mg/kg per day. The liposomal formulation of amphotericin B stays intact in the bloodstream and has been found to be present in all internal organs after administration by I.V., including the central nervous system [143,158]. The recommended dosage of liposomal AmB is between 3 and 5 mg/kg per day [143]. Two-thirds of deoxycholate AmB is excreted intact by urine and feces, and over 90% is excreted within 1 week of treatment, which suggests that elimination of this formulation of AmB is not dependent on metabolism. In contrast, only 10% or less of liposomal AmB is excreted intact [158]. The elimination half-life of AmB has been determined to be 15 days, with disappearance from the serum and urine observed after 3–7 weeks. This indicates that AmB is stored in body tissues and slowly released and eliminated after cessation of treatment [159,160].

#### 2.4.4. Side Effects and Human Targets

Many of the macrolide antifungal compounds discussed in this manuscript have nonspecific targets, including some types of human and other mammalian cells. Since amphotericin B is one of the most widely used antifungal drugs, its actions on human cells have been studied extensively, and it will serve as the model polyene antifungal in this review. The conventional D-AmB micellar formulation has been shown to have intense side effects, including hemolysis, vomiting, fevers, hypotension, and nephrotoxicity, amongst others [156,161,162]. AmB has been shown to be cytotoxic to human colon epithelial cells via binding to the plasma membrane and being endocytosed into the cells as a defense mechanism [163]. The effects of AmB on fungal cells are dependent upon the higher affinity of AmB to ergosterol than cholesterol. However, due to the similarity between the two sterols, AmB dimers can still bind cholesterol and cause the formation of transmembrane channels, which leads to leakage of ions from mammalian cells, abnormal electrolyte balance, and decrease in ATP production [140]. It is speculated that lipid-based formulations of AmB are less toxic to human cells when compared to conventional deoxycholate formulations, as they prevent intracellular trafficking of AmB by endosomes. Free AmB perforates through the membrane and is trafficked into the cell through endocytosis. Kagan et al. showed that the conjugation of AmB to arabinogalactan reduced the perforation effects seen with free AmB in mammalian cells, but still allowed penetration of fungal cells, making the conjugated formulation an attractive option for therapeutics [164]. Indeed, many research groups have developed lipid-conjugated or liposome-incorporated AmB (and other polyenes including nystatin) as a way to safely deliver the drug; however, these formulations often come at a high cost and require a higher dose to be effective [140,165,166]. In addition to cytotoxic effects, the presence of AmB induces a proinflammatory response of interleukin 1β (IL-1β), tumor necrosis factor α, and nitric oxide (NO) production [161,164].

### 2.5. Immunosuppressants

The macrolide immunosuppressants represent a more recently discovered class of drugs that are used to prevent organ transplant rejection and treat inflammatory skin diseases [167]. Tacrolimus is an immunosuppressant that was discovered in 1984 in *Streptomyces tsukabaensis* cultures found from soil near Tsukuba, Japan [168,169]. It has been used to prevent kidney, liver, and bone marrow rejection in transplant patients [169,170,171]. A topical formulation of tacrolimus is the first non-glucocorticoid shown to be effective at treating atopic dermatitis [170]. Pimecrolimus is an ascomycin derivative that was developed specifically to treat inflammatory skin conditions, including psoriasis and atopic dermatitis [172,173]. Rapamycin, known by the common name sirolimus, was isolated from the actinomycete *Streptomyces hygroscopicus* from soil found on Easter Island, where it was first determined to display antifungal properties [174,175,176,177,178]. It was later found to display immunosuppressive properties, and has been developed for the prevention of renal, pancreatic islet cell, liver, and heart transplant rejection [174,179].

#### 2.5.1. Structures

Tacrolimus and pimecrolimus are very similar 23-membered macrolide lactones, with the only structural difference being two chemical side groups on pimecrolimus that are not present on tacrolimus [169,170,180]. Tacrolimus is neutrally charged and hydrophobic [171], while pimecrolimus is up to 20 times more lipophilic than tacrolimus, likely due to its side groups [173,180]. Sirolimus is a 31-membered macrolide that displays high lipophilicity and is insoluble in water [174,181]. A summary of the immunosuppressant macrolides can be found in Table 5.

#### 2.5.2. Mechanism of Action

##### Inhibition of Calcineurin

Presumably due to their similar structures, tacrolimus and pimecrolimus display a similar immunosuppressive mechanism of action. In 1991, Liu et al. showed that tacrolimus targets the calcium-activated phosphatase calcineurin [182]. This was noted to be the same ultimate target as cyclosporine, a drug that was developed earlier than tacrolimus for the treatment of inflammatory skin disorders [170,182]. Calcineurin regulates the translocation of nuclear factors responsible for the regulation of promoter activities of mRNA transcription mediators [173]. Tacrolimus and pimecrolimus each bind to the immunophilin macrophilin-12, and the complex can then bind calcineurin, inhibiting its ability to dephosphorylate certain transcription factors, including nuclear factor of activated T cells (NF-AT) [171,172,173,183,184,185]. NF-AT is responsible for the mRNA transcription of the inflammatory cytokines IL-2, IFN-α, IL-4, and IL-10, which are blocked during treatment with pimecrolimus or tacrolimus [173]. Interestingly, the inhibition of calcineurin is dependent on the drug being in complex with its intracellular receptor [170]. The final result of this mechanism in the immune system is suppression of T-cell activation [169,173].

##### Inhibition of the Mammalian Target of Rapamycin (mTOR)

Sirolimus, also called rapamycin, binds to the intracellular receptor FK-binding protein. The complex has no effect in calcineurin, but instead goes on to bind and allosterically inhibit the activation of mTOR, a regulatory protein responsible for the coordination of movement from the late G1 to S phase of the cell cycle. This inhibition stops T-cell proliferation by suppressing the cytokines IL-2, IL-4, and IL-15 [174,175,176,179]. Sirolimus inhibits T-cell proliferation at a later stage in the cell cycle when compared to the calcineurin inhibitors [179]. One of the mTOR complexes, mTORC1, which is frequently impaired in certain cancers, has been shown to be more sensitive to treatment with sirolimus than mTORC2, making sirolimus a potential anticancer treatment as well [174].

#### 2.5.3. Pharmacology

Oral administration of tacrolimus results in peak blood concentrations from 1 to 2 h later, indicating rapid absorption, which can be delayed in some transplant patients that have altered gut motility. The bioavailability of tacrolimus ranges greatly, between 4 and 89% in kidney and liver transplant patients. In healthy subjects, the bioavailability is between 14 to 17%, a discrepancy which is not yet understood. Lower bioavailability is associated with hepatic metabolism via cytochrome P450 3A4 isozyme and potentially P-glycoprotein efflux pumping at the luminal face of enterocytes. Tacrolimus displays a concentration-dependent binding to erythrocytes. It does not significantly bind to lipoproteins in serum, but rather binds to α1-acid glycoprotein and albumin. Elimination of oral tacrolimus occurs 75% through biliary excretion, with an elimination half-life of about 32 h, though this varies between patients [169,171,184,185].

Pimecrolimus also displays rapid absorption after oral administration, typically between 0.7 and 1.4 h for a single dose, and between 0.8 and 2 h for multiple doses [186]. The distribution of pimecrolimus within the blood is concentration-dependent, with higher concentrations of pimecrolimus resulting in higher percentages of the drug being found in plasma versus blood cells. When tested against 10 different P450 isozymes, pimecrolimus was metabolized primarily by the CYP3A4 isozyme, similarly to tacrolimus. Radioactively labeled pimecrolimus was found to be primarily excreted within the feces, with only minor amounts being excreted through the urine [187]. 

Sirolimus is often taken orally in combination with ciclosporin once per day, where administration results in rapid absorption into the blood, with peak levels observed about 1 h after the dose was given. The bioavailability of sirolimus, again when taken concomitantly with ciclosporin, is only about 15%, which is attributed to hepatic metabolism by cytochrome P450 3A4 isozymes and to countertransport by intestinal P-glycoprotein multidrug efflux pumps. Sirolimus is sequestered by red blood cells up to 94.5%, which is believed to be in part due to the cells’ immunophilin content. The fecal and biliary pathways are thought to be the primary elimination routes, with an elimination half-life of 62 h [174,179,181].

#### 2.5.4. Side Effects and Human Targets

While the majority of side effects of the macrolide immunosuppressants are mild, some can be more intrusive [167]. Since calcineurin is a phosphatase involved in many cellular processes, systemic dosage of the calcineurin inhibitors can cause significant effects in the human body. These effects are typically dependent on dose, concentrations in the individual’s blood, and how long the drugs are taken. The significant side effects are hypertension, hyperglycemia, nephrotoxicity, hyperlipidemia, too much immunosuppression, and psychiatric effects [170,184,185]. Renal side effects are proposed to be due to the increase of renal vascular resistance, reduction of glomerular filtration rate, and the synthesis of endothelins, which are all likely a result of the calcineurin-inhibiting properties of tacrolimus [169]. Topically applied tacrolimus does not appear to cause these intense side effects, but rather may cause local site burning or itching sensations on the skin. Only about 0.5% of topically applied tacrolimus penetrates the skin deep enough to be absorbed into the blood, making the symptoms associated with systemic administration uncommon [170]. Indeed, Billich et al. compared the skin penetration abilities of pimecrolimus, tacrolimus, and commonly used topical glucocorticosteroids. Pimecrolimus showed a lower penetration rate by a factor of 70–110 when compared to the steroids, and a rate 9–10 times lower than that of tacrolimus, indicating that systemic circulation after the topical application of pimecrolimus is significantly less common than with the glucocorticosteroids [180]. 

Common side effects of sirolimus include dose-dependent hyperlipidemia and cytopenias including anemia. Unlike the calcineurin inhibitors, nephrotoxicity is uncommon with the use of sirolimus [175,176]. Since these effects are dose-dependent, some have proposed that sirolimus levels should be monitored periodically over the course of treatment, since individual excretion rates of the drug are variable [179].

## 3. Conclusions

Macrolides, well known for their cytotoxic activity, also display a variety of beneficial bioactivities that are important to human health. Some toxins have themselves been shown to display anticancer and antimicrobial properties. The macrolide antibiotics include one of the most influential antibiotics discovered to date: erythromycin. While some macrolide antibiotics are limited in their use as pharmaceuticals due to poor pharmacokinetics and/or adverse side effects, synthetic blends have been able to overcome these issues. The antiparasitic macrolide ivermectin has virtually halted transmission of onchocerciasis, and has thus been deemed a “wonder drug”. Macrolide antivirals have been effective in the treatment of HIV, herpes simplex type I, SARS-CoV-2, influenza, and more. Amphotericin B is on the WHO’s *List of Essential Medicines* for its efficacy in treating systemic fungal infections. Immunosuppressant macrolides have been successfully used to prevent organ transplant rejection and treat atopic dermatitis.

The wide range of bioactivities found within the macrolide group highlights the importance of drug discovery. Some compounds may originally be discovered as toxins, but later found to have therapeutic applications. It is important to study the mechanism of toxicity in order to reveal potential therapeutic applications. For example, many macrolide toxins target the cytoskeleton in order to disrupt cellular processes. This mechanism of action has been manipulated to target cancer cells, and a derivative of the toxin halichondrin B has been approved by the FDA for the treatment of certain cancers. Without the discovery and investigation of marine toxin compounds, this life-saving drug would not be available today.

Certain macrolide molecules display biomimetic properties due to their chemical structures, which allow them to bind to and interact with components of human cells. This review highlights some of the important functions macrolides have played in the preservation of human health, from the treatment of viral, bacterial, and fungal infections, to the near-eradication of the parasitic disease onchocerciasis, to supporting successful organ transplants and treating cancer; the applications of macrolides have had a profound impact on us as human beings. It can only be speculated how many other potentially beneficial functions have yet to be discovered within the macrolide class.

## Figures and Tables

**Table 1:** Structure of Macrolide Toxins.

Macrolide Class	Compounds	Source *	Example Structure
Trisoxazole-ring-containing macrolides	Kabiramides Ulapualides Halichondramide Jaspisamides Mycalolides	*Hexabranchus* sp. *Hexabranchus sanguineus* *Chondrosia corticata* *Jaspis* sp. *Mycale* sp.	**Kabiramide C** 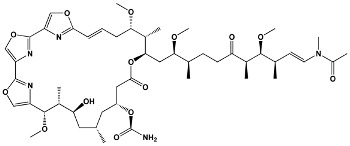
Macrodiolides	Swinholides Ankaraholides	*Theonella swinhoei* *Geitlerinema* sp.	**Swinholide A** 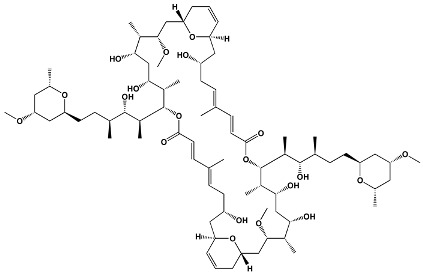
Diterpene macrolactones	Reidispongiolides Sphinxolides	*Reidispongia coreulea* *Neosiphonia superstes*	**Reidispongiolide A** 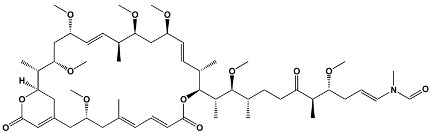
Other macrolides	Jasplakinolides Latrunculins Spongistatin Aplyronine Venturicidins Mycolactone Halichondrins	*Jaspis johnstoni* *Latrunculia magnifica* *Hyrtios erecta* *Aplysia kurodai* *Streptomyces* sp. *Mycobacterium ulcerans* *Halichondria okadai*	**Jasplakinolide** 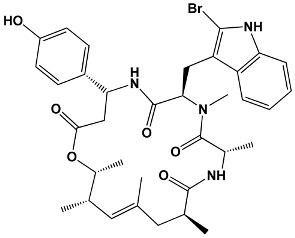

* Toxins may be produced by multiple species; one representative example is provided.

**Table 2:** Structures of Macrolide Antibiotics.

Members in Ring	Compounds	Source	Example Structure
12	Methymycin	*Streptomyces venezuelae*	**Methymycin** 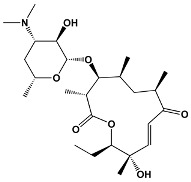
14	Erythromycin Pikromycin Oleandomycin	*Saccharopolyspora erythraea* *Streptomyces venezuelae* *Streptomyces antibioticus*	**Erythromycin** 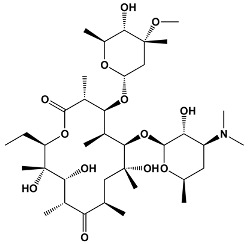
15	Azithromycin	Erythromycin derivative	**Azithromycin** 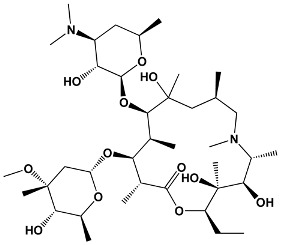
16	Spiramycin Tylosin Josamycin	*Streptomyces ambofaciens* *Streptomyces fradiae* *Streptomyces narbonensis*	**Josamycin** 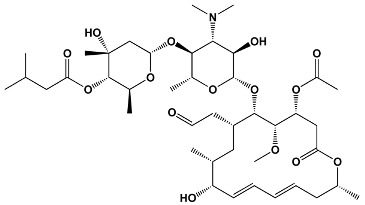

**Table 3:** Structure of Macrolide Antivirals and Antiparasitics.

Macrolide Class	Compounds	Source	Example Structure
Avermectins	Ivermectin Selamectin Monoxidectin Abamectin	Avermectins are produced by *Streptomyces avermitilis*; specific compounds are derivatives	**22,23-dihydroavermectin B1a** 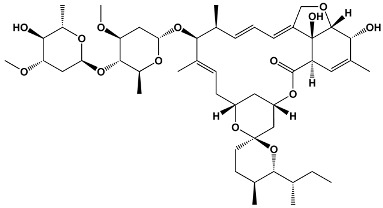
Plecomacrolides	Balticolid	*Ascomycetes* spp.	**Balticolid** 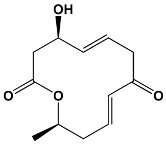
Oxygenated macrocyclic lactones	Bryostatins	*Bugula neritina*	**Bryostatin 1** 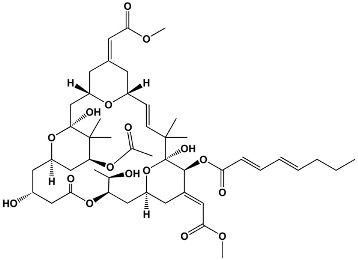

**Table 4:** Structure of Macrolide Antifungals.

Macrolide Class	Compounds	Source	Example Structure
Polyene	Amphotericin B Nystatin Natamycin Filipin	*Streptomyces nodosus* *Streptomyces noursei* *Streptomyces natalensis* *Streptomyces filipinesis*	**Amphotericin B** 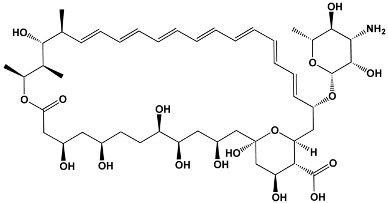
Salicylate	Cruentaren A	*Byssovorax cruenta*	**Cruentaren A** 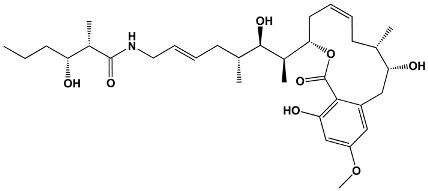

**Table 5:** Structure of Macrolide Immunosuppressants.

Members in Ring	Compounds	Source	Example Structure
23	Tacrolimus Pimecrolimus	*Streptomyces tsukabaensis* Ascomycin derivative	**Tacrolimus** 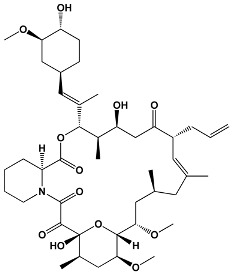
31	Sirolimus (Rapamycin)	*Streptomyces hygroscopicus*	**Sirolimus (Rapamycin)** 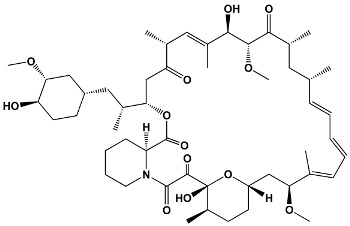

## Data Availability

Data sharing not applicable.
